# Observations on Cancer

**Published:** 1819-06

**Authors:** T. Parkinson


*For the London Medical and Physical Journal.*

### *Observations on Cancer;* by T. Parkinson, M.D.

IN the following observations I purpose to treat of cancer, distinctly, under two different stages:—1. The scirrhous stage, *Symphylakesis passiva;* 2. The cancerous stage, *Symphylakesis progressiva.*

I have opposed the term Symphylakesis to that of Cancer; and, in rendering the term, the identity of the disease will be displayed.

Symphylakesis—Συν-*cum*, with, *Φυλασσω, incarcero,* to imprison: *complicated incarceration.*

Symphylakesis is, and cannot be otherwise, a secondary disease, arising out of one of those processes by which inflammation retires; namely, *Proscollèsis*, or morbid accretion. But, as the term implies, it has a peculiarity characteristic of its very nature, and by which it is distinguished from all other diseases, bearing, in external appearance, a strong resemblance. I am called upon, then, to point out such peculiarity. This I hope to be able to do most clearly, in giving the analysis of the disease.

I have deemed it expedient and necessary to banish altogether the hypothetical terms as applied to this disease, *hereditary*, *predisposition*, *idiosyncrasy*, *cancerous virus*, &c. and confine myself to the mere narration of the morbid animal process by which the disease is originally formed, and the manner in which it proceeds to its fatal termination; not deduced from theoretical notions, but from an abundance of practical observation.

It has fallen to my lot to be very generally consulted, both at home and at considerable distances from home, in cases supposed to be cancerous; in many of which I have had recourse to the operation, or, in a greater number of cases, have superintended the operation when it has been performed by others. This has afforded me opportunities sufficiently numerous of examining the diseased parts, after they have been removed by the operation; and in all instances I have discovered, that the disease called cancer consists of what the term Symphylakesis implies, namely, complicated incarceration.

Complicated incarceration, stands opposed to simple incarceration; simple incarceration, signifying merely the imprisonment of any animal solid, by being surrounded by accreted adipose membrane, without vital connexion and intertexture; and, consequently, without any new function being set up between the incarcerating cyst and the substance incarcerated; instanced in steatomatous tumors and scrofulous glandular tumors: and this form of disease is expressed by the term Phylakesis, *Φυλασσω, to imprison.* But complicated incarceration signifies something more than what i discovered in steatomatous and glandular tumors of the scrofulous denomination, namely, an association of a vital nature, connexion, and intertexture; and, consequently, a new and adscititious function set up and established between the cyst and the body enclosed by it: this is instanced in the scirrhous tumor denominated Symphylakesis.

The solids so morbidly associated are generally supposed to be gland and adipose membrane: this is the common association in the disease before us, but perhaps not the only one. This circumstance will, however, be enquired into, when we consider that disease which is usually denominated Fungus Hæmatodes.

In the present discussion, I shall limit myself to that particular disease which is denominated the Cancerous Scirrhus; and which, if uninterrupted, destroys life through the slow and painful process of gangrenous ulceration, and the consequent constitutional sufferings. I shall, therefore, take it for granted, that the morbid connexion and function are established between a gland and adipose membrane.

Now, no such connexion can be formed in a healthy state of parts; while in health, they will each be distinct in their anatomical construction, and will duly exercise their distinct and individual functions: consequently, will be obedient to those laws which govern life, health, and disease, in legitimate solids.

But, when a change of structure takes place from disease, a corresponding change of function also will be the inevitable consequence. Under these circumstances, the subjects of disease will not be governed by the laws of health; neither will they obey those which are rigidly observed under all the primary diseases, in which there is no change of structure; and, consequently, no change of function, except what consists in an increased or diminished exercise of it.

The manner in which this change of structure, and of function, is effected, becomes our next consideration.

The two originally distinct solids, adipose membrane, and gland, become morbidly associated in their substances and in their official capacities: hence, they are no longer legitimate in their nature; neither are they under the control of the laws of the animal economy.

It appears to me essential to the accomplishment of this association, that the gland and the adipose membrane be both under inflammation at the same time, and that they be kept in close contact, so that accretion of the two solids may be fully and firmly established; and, indeed, that they should pervade each other's substance, or so intimately mix, as to become one individual adscititious solid.

The construction of the breasts of women is favourable to this kind of association, they being composed of glandular and adipose substances, in some degree connected with each other, but yet distinct in their structural arrangements and functions; and they require only a less intensity of inflammation than what is requisite to destroy the solids; but yet sufficient to produce adhesion, and that the same tone of inflammation be kept up for some time, to unite them in one adscititious substance.

It appears, then, that Symphylakesis is always a secondary affection, the resulting consequence of a certain intensity of inflammation remaining after the parent inflammation has ceased; and cannot have existence without inflammation as its cause. Hence, then, is manifest, the necessity of proper attention to those diseases which prevail in the breasts of women, from the approach of puberty till the final cessation of the menses. It is possible, that a slight tone of inflammation, if suffered to be long continued, may be productive, ultimately, of the most serious and fatal consequences.

An enquiry into the nature of those changes which take place in the breasts of girls, and the function they assume at puberty, and preserve till the approach of old age, together with the manner which, at that period, they are deprived of their function; will direct us in drawing our nosological deductions, and lead us to the most appropriate treatment of those diseases to which they are particularly liable.

In childhood, the breasts of girls are not distinguishable by any external marks or configuration from those of boys; but, at puberty, those of the female have to prepare for the performance of a peculiar office they are expected to be called upon to fulfil; that is, to provide milk for future offspring. In this preparatory process, it is remarked, that the breasts enlarge, and become prominent, in consequence of assuming a new organization at developing what had been hitherto concealed; namely, a glandular substance, with an additional accumulation of adipose membrane: and, indeed, such an accretion seems essential to afford them their official capacity. In this state they remain, with those varieties which depend upon pregnancy, menstruation, giving of suck, &c. till the approach of old age; and then it is, that the functions not only of the breasts, but those of all the female sexual organs, cease; manifested by the incapability of bearing children, the final cessation of the menses, and by the removal of the glandular substance of the breasts, by a process retrogressive of that which established it at puberty.

Now, the breasts of women are not only exposed to injuries from external violence, which are followed by inflammation, but they are also peculiarly liable to inflame, under those changes which they undergo at the approach of puberty, in the intervals of menstruation, during that process, in pregnancy, in giving suck, in weaning; and, in that important change which always take place at the final cessation of the menses.

At the approach of puberty, it has been already observed, that new appearances, properties, and capabilities, are put on; and, on this occasion, there is, perhaps always, some degree of pain complained of, at least generally there is: and it is not at all uncommon for that pain to be severe, especially on the approach of every periodical visitation, attended with enlargement of the whole substance of the breast. These symptoms, however, are mitigated, or even vanish, on a free menstrual evacuation, until the near approach of the succeeding visitation. This affection is seldom attended with any very serious consequences: so soon as the habit of menstruation is fully established, the complaint ceases altogether.

But there is another morbid affection, which sometimes occurs at puberty, in the acquisition of official capacity, which is more deleterious in its consequences; and to which I would lay claim to your close attention. Instead of the whole of the glandular substance being enlarged and painful, a certain definable part of it only is so affected, somewhat painful, especially under pressure; and is readily distinguished from the sound parts of the breast by its extraordinary hardness or density, forming a tumor describable by the feel. It does not resolve, though it is less painful in the intervals of menstruation.

I have mentioned this affection as occurring at puberty; it nevertheless happens, from adequate causes, at any period between the first appearance and the final cessation of the menses: and, let me add, the adequate causes are, whatever are capable of exciting and keeping up a certain, but low, tone of inflammation, in some distinct part of the glandular substance of the breast and the adipose membrane surrounding it.

A disease of this kind, being formed in early life, will remain dormant, and seemingly innoxious, without causing the woman to complain; will be passive, even when the other parts of the glandular substance are in the full exercise of their function: but it must be remembered, that, at the approach of old age, the peculiar functions of the breasts are to cease wholly, and that is effected by the removal of that organic substance which was put on at puberty, and in which the official capability resided. All this is easily, and is always, accomplished under a natural and healthy state of the breasts; that is, the glandular substance of the breasts is absorbed, leaving the integuments loose and flabby, or more commonly filled with adipose substance. But, under a diseased state of the adipose membrane, and especially under that form which I have described as accretion of adipose membrane, converted, as it were, into ligament, enclosing a part of the glandular substance, united with it in structure and function; an illegitimate substance, disobedient to the laws of the animal economy; it will be readily allowed, that such a production will not so easily be removed: indeed, it cannot; but that process which does actually remove the sound parts, makes the same attempt upon those diseased, and rouses them into deleterious energy: that is, inflames them. They then exercise their own adscititious function, establishing a system of their own; whose basis is morbid accretion and vital connexion between solids of every description, even embracing the bones.

It requires no extraordinary share of attention and discernment, to discover that the whole of this process is actually progressive inflammation, and should have some influence in directing the method of treatment in the scirrhous stage, or whilst it remains *Symphylakesis passiva.*

From this view of the subject, then, it appears that the scirrhus originates in inflammation; that it remains dormant, as a disease, after inflammation has ceased; at an advanced period of life, it is provoked into action of the same kind, and to the same effect, as that with which it was originally formed; that its progress is accretion, and all its consectaria; that it is not under the government of the laws of natural solids; that its type becomes *progressive;* and that its issue is fatal.

Having traced the scirrhus from its beginning, through its passive stage, down to its cancerous stage, I shall now view it in its progressive type, that is, as *Symphylakesis progressiva.*

What I would have understood by the progressive type, is, that state of the scirrhus which is advancing fast towards ulceration, or is already ulcerated. This state, though not peculiar to advanced age, rarely is met with till towards the approach of the final cessation of the menses. It then, after having been in a quiescent state for many years, is suddenly roused into strong and rapid action. The identity of disease is the same in every period of its existence; that of accretion of one solid to another, with vital connexion established between them: and, although the process commenced with the adipose membrane and the gland, yet it extends to all solids whatever, the skin, muscles, bones, &c.; embracing them all in one solid mass of disease, extending in every direction. The axillary glands become scirrhous, and very commonly the arm on that side is œdematous; the surface of the original tumor becomes ulcerated; the pain becomes severe and lancinating; and the constitutional sufferings are obviously increased. The progress of the cancerous ulceration is peculiar to itself: whilst it increases in all directions, it becomes gangrenous on its ulcerated surface; of course, the neighbouring parts are progressively brought into a diseased, a scirrhous, state, and constantly dying by gangrene: consequently, the discharge from the ulcerated surface is highly fœtid, and the constitutional sufferings or sympathies are truly afflicting.

There is, however, a disease of the breasts of women much resembling Symphylakesis, particularly in external appearances and impenetrable hardness, of a character and nature very different. It affects the breast during giving suck, and not unfrequently at a remote period of that process, be the age of the subject of it what it may.

The disease I allude to, is Phylakesis; or simply, incarceration of a gland, or a number of glands, by accreted adipose membrane, without vital connexion and associated function being established. It generally terminates favorably, by the incarcerating cysts being brought into a state of gangrene by the constant application of cold, and the glandular substance afterwards running into small distinct abscesses, which heal kindly after a few days: and especially if the woman cease to give suck from that breast.

I have been consulted in a great number of cases of this kind; and have always been able to distinguish them from what is called legitimate cancer, chiefly by the unevenness of surface intimating that it is not any determinate portion of the glandular substance which is diseased, but the whole of it, exhibiting distinct prominences under the skin, without any change of colour of the integuments in the beginning, but increasing in prominence, and gradually acquiring a livid hue, on the external surfaces. Lancinating pains are complained of, resembling those so common in the cancer. I have always employed the same means with the same effect, —success; and shall, therefore, mention what that treatment consists of:—℞. Extract Conij. ʒij.; Aquæ fontan. ℥viij. M ft. Lotio, partibus affectis assidùe applicanda. The bowels to be kept rather open with sulphate of magnesia, or any other cooling aperient. The worst cases of this kind, have got quite well in about six weeks after the cold lotion has been had recourse to.

Now, I consider the disease I have just described, to be precisely the same as the scrofulous glands which prevail in the neck. I repeat, simple incarceration of a gland by its surrounding accreted adipose substance, and the success I have met with in the treatment of it, in the breasts of women, has naturally led me to the adoption of the same means, when the disease has been resident in other parts of the body. The effects have been similar, though more slow, in progress: that is, the cyst being destroyed by cold, which is easily accomplished either by the means above-mentioned, or by the generation of more intense cold, if necessary, by the evaporation of spirit of wine or alcohol from the surface of the tumor; then the gland will have the opportunity of suppurating and healing, or of being reduced to its natural size by the process of absorption, and the healthy exercise of the neighbouring parts upon it.

But, to return to the disease professedly the subject of this essay, Symphylakesis. When the disease is once formed in early life, it may, and very often does, give little trouble, and is disregarded, and that even for many years; but, at a certain period, in the way already pointed out, it is roused into deleterious energy: it rages with almost uncontrolable fury, and consumes its victims by the extremes of protracted torture. It is a matter of deep interest, then, to discover, at least, what constitutes its real character; and, if possible, to obtain a method of treatment, if not altogether adequate to the removal of the disease, when the knife has been too long rejected, yet capable of mitigating that sum of human misery which is so eminently connected with the cancer.

Viewed in the light I have shewn it, I am persuaded there cannot be any conflicting opinions with respect to the means to be had recourse to, when the disease is once known to be instituted. It appears, from what has been stated, that it commences with inflammation of a gland, and a portion of the adipose membrane immediately investing it; that the primary disease, in the act of retiring, by one of those natural processes by which it is known to retire—adhesion, leaves a morbid connexion and adscititious function, set up between the two substances implicated in the disease; and this latter is a secondary affection, the offspring of a primary one, remaining after its parent has ceased to be. Now, there is no means, with which I am acquainted, which can be employed with any reasonable prospect of overcoming this morbid connexion and function, and of averting that misery of which it is the precursor, excepting the actual separation of one of the morbidly-united parts from the other, or the total removal of them altogether from surrounding healthy parts.

The first of these operations appears, from what has been said, to be next to impossible; and, indeed, were it accomplishable, there is no certain test by which it can be ascertained that it is actually effected: and the very nature of the disease points out, what daily observation fully confirms, that any operation, not extended beyond the limits of the diseased solids, will but accelerate the progress of the malady, and aggravate the sufferings of the subject of it. An attempt, then, to effectually and certainly separate the two accreted solids, must yield to the less painful, but more decided and efficacious, operation, of the total separation of the diseased parts altogether from those which are healthy.

Certain various opinions have been advanced by the advocates for the operation last-mentioned, respecting the proper period of the disease for the performance of it. A review of the process by which the disease is originally formed, and by which also it makes its progress through all its stages, will, I am inclined to believe, reconcile any conflicting opinions which prevail, perhaps at the present time, on that question. I would, therefore, recommend a steady and careful examination of the doctrine I have promulgated respecting the nature of the cancer; and, if it be found to be built upon solid and just principles, it will unite all in one consolidated opinion, that the most proper time to perform the operation is, so soon as the morbid process, Symphylakesis, is known to be set up: For what can be the use of delay, when the progress of the disease is identically the same process as that which first formed it, namely, accretion?

A supposition that the cancer was produced by a peculiar poison, would naturally lead to, and in some degree justify, the conclusion, that the operation ought to be delayed until it could, with some degree of precision, be ascertained how far the poison had extended its influence; and, therefore, to what limits the operation should be carried. This, however, is not strictly correct; for, if the disease makes progress by the extension of poison, there cannot be any greater certainty respecting the distance it has extended to, after many years' delay, than there was when the disease was first discovered. And so also it is, if the accreting process be admitted as the basis of the cancer, I cannot perceive what advantage can be obtained from delaying the operation any longer than till its true character is understood.

Various methods of treatment have been recommended, approved, adopted; and all, in their turns, rejected: and those, even now applauded, will probably share the same fate, excepting, however, those which have for their bases the total removal of the diseased parts from those which are sound.

The comparative merits of the knife and the caustic are pretty fully determined, and properly in favour of the former; therefore, it is unnecessary for me to dwell upon that subject. I would, however, throw into the scale an observation, which strikes me as being of some practical importance. As the parent of the disease, (that is, the primary affection which produced it as one of its offspring,) was *inflammation* resolving by accretion; that, so long as a certain tone of inflammation continues, the accretion will extend, but no longer. Hence it is that, when the disease is formed in early life, it remains passive until the approach of old age; unless, by some casual circumstance, a new accession of inflammation should be provoked. But, let it be remembered, that, at an advanced period of life, the function of the glandular substance of the breast must be abolished, and that by the removal of the glandular substance, by an animal process retrogressive, which first deposited it. This process, although fully adequate to the removal of the healthy substance, cannot be so successful with an association of solids with unnatural function, which constitutes the scirrhus: the attempt, however, has the power, and indeed constantly the effect, of provoking the diseased association into diseased energy; into a slight tone of inflammation; under which state, and under that state only, can the accreting process make advances: consequently, it is only under an inflammatory action that the disease called scirrhus, or cancer, can make any progress.

How plain is it, then, that the primary disease is inflammation; that the secondary affection, is a morbid animal process, set up in the resolution of inflammation; that the progress of the cancer is inflammatory; and that the subject of it is, such portion of the associated glandular and adipose substances by morbid accretion, which cannot be removed by the ordinary animal processes, before the approach of the final cessation of the menses. Not that it is supposed that the scirrhus cannot be brought into an inveterate and ulcerated state before the period of life just alluded to: it often is; nor is it at all more surprising, that the removal of the glandular substance of the breast is sometimes attempted, and even accomplished, in the most vigorous and healthy periods of life: And let me ask, are not these concomitant and simultaneous in all cases where the scirrhus is roused into deleterious energy in early life?

The following inference is deduced from the foregoing premises: that the disease, called cancer, in the breasts of women, is morbid accretion; and the precise subjects of it, are those portions of the breast which cannot be removed by a natural process, at the approach of old age, in common with the sound parts, in consequence of their morbid change of structure and function, effected by the process of accretion.

Innumerable, almost, are the articles of the materia medica, and other substances, which are comprehended in the catalogue of specific remedies or antidotes; that is, those which have the property of destroying, or of expelling, the cancerous poison: and some of them have a most deleterious tendency, such as arsenic, oxydes of hydrargyrus, cicuta, &c. &c. These, and others of no less powerful action on the living animal solids, and, consequently, capable of altering the state of the animal and vital actions, may be tried with safety, under the immediate superintendance of a skilful director; but, the moment he proclaims his success, though that is only delusive, the ignorant will not fail to imitate him, and endeavour to surpass him by venturing to employ the remedy with greater force and frequency (perhaps to the destruction of the deluded patient) than the original recommender dared himself to have done.

The point, I believe, is pretty well settled, respecting a specific virus being the cause and production of the cancer; and that of its hereditary character, appears fast retiring into the back-ground of popular opinion. But, how much soever be our acquirement of the knowledge of the real nature of the cancer, little has been discovered of late years which holds up a distant hope of pointing out any remedy, excepting that of removing the diseased from the sound parts.

Many years ago, when Mr. Howard was surgeon, and Dr. H. Vaughan (now Sir Henry Halford) physician, if I mistake not, to the Middlesex Hospital, and had principally the direction of the treatment of cancerous patients, for the reception of which one of the wards of the hospital was appropriated, I had several communications with Sir Henry. I believe, I sent him the history of several cases supposed to be cancerous, and deemed such by his father, then an eminent physician at Leicester, as well as by others of the profession, which yielded to the external application of a cold cicuta lotion.* And I must add, that the diseases to which the lotion had been successfully applied, were of the character which I have fully described;† and to which, when I had distinguished it from the cancerous scirrhus, and had obtained the means of always correctly distinguishing them, I gave the name of *Scirrhus suppurans;* shewing its

* Vide page 473. † Ibid.

resemblance to the cancerous scirrhus in hardness and want of sensibility, but differing from it in its progress and mode of termination. Professing now, however, to denominate diseases from their identities, I have given it the appellation *Phylakesis.*

I shall here take the liberty of introducing a case in point. Mrs. Hubbard, of Countesthorpe, about seven miles from Leicester, a healthy young woman, in an early period of suckling her first child, was attacked with a disease in her breast, which increased in extent, though not in pain, becoming daily harder under the application of poultices and other topical applications, chiefly recommended by her neighbours. At length, finding no benefit, she became apprehensive of evil consequences, and consulted a physician at Leicester. After the application of leeches, and the use of aperients, &c. for some time, the Doctor declared it to be a cancer, recommending, as the only certain remedy, the total removal of the breast. The patient consented: lodgings were provided in Leicester, a surgeon fixed upon to perform the operation, and the time appointed. In the meantime, Mrs. Hubbard called upon a lady in Leicester, who, she heard, had undergone an operation similar to that which she was about to submit to: indeed, I performed it. The lady advised her to consult me, previous to being operated upon: she did so, and I immediately discovered it to be phylakesis, not symphylakesis. I declared my opinion, and proposed a consultation with the Doctor; to which he objected, on the alleged grounds that there could not be two opinions on the case. The patient, however, put off the operation, and placed herself under my care. I, of course, employed the means I have described; and the event was, she recovered in the way and manner as all other such like diseases have done.

Pressure, as a remedy for the removal of the cancerous scirrhus, has obtained some fashion; and, perhaps, when it can be employed so as to embrace the whole of the diseased parts, and completely cut off all their connexion with the sound ones, and at once destroy the function of diseased solids, it might be successful; and yet not so formidable an operation as that by the knife; and would, in many instances, obtain a preference. But, if it fall short of so much efficiency, I should really be under serious apprehension that it would prove an accelerator of the disease, by exciting inflammation.

I may, however, venture a suggestion, with which I shall conclude this paper. Reduce the general animal and vital actions very low; and then destroy the diseased parts by intense cold, so long continued as to prevent re-action, which may be accomplished by the evaporation of alcohol from the surface.

*26, Falcon-square; Jan.* 12, 1819.

